# Supplementary material for: Prevalence and determinants of depression and/or anxiety among adults using Kenya Demographic and Health Survey of 2022: Multilevel logistic regression analysis
Source: PLoS One. 2025 Mar 28;20(3):e0319571. doi: 10.1371/journal.pone.0319571 (PMC11952211; doi:10.1371/journal.pone.0319571)
Supplement: Attachment — Submitted filename: Reviewer comments_plosOne.docx [file pone.0319571.s001.docx]

As the reviewer, I have conducted a detailed assessment of the manuscript titled "Determinants of depression and/or anxiety among the adult community in Kenya: Multilevel analysis of Kenya Demographic Health Survey of 2022" using the STROBE Statement checklist for observational studies. Here is an in-depth report addressing each of the checklist items and providing specific line references where issues have been identified:

**Title and Abstract:**

1. **(a)** The title accurately indicates the study's design as a multilevel analysis of kenya demographic health survey of 2022.

- But it was also better to put the specific statistical model like multilevel binary logistic regression analysis as a study design.

**(b)** The abstract did not effectively describe what was indicated the title.

- The study revealed the overall prevalence of depression and/or anxiety among the community in Kenya from Kenya Demographic and Health Survey data of 2022, but the title you indicated and the objective you described in the abstract are completely unrelated. So, how do you relate the title with that of line 29-30?
- Line 32-33 needs to be rewrite.
- Line 33-34 is better suited for only multilevel analysis, as it’s reasoned for both Multilevel and logistic regression is inappropriate. Because the clustering nature of the data is not a reason for both multilevel and binary logistic regression analysis.
- Line 35 “The overall prevalence of 3.84% with a 95% CI of (3.56, 4.14)”, Whose prevalence, is it? It needs to be corrected.
- Line 36 “In multivariant multilevel logistic regression”, what does that mean multivariant? How do you relate with the word “multivariate” and “multivariable”.
- The keywords (line 45) you indicated is not enough.
- Overall, the abstract is not effectively described what was done.

**Introduction:**

2. The introduction section presents several issues:
   - Line 54 and line 60: DSM-5 and WHO need to be abbreviated at first.
   - Line 91-98: font size is not similar with others.
   - Line 97-98: “This study aims to determine the associated factors of depression and anxiety with their burden among adult communities in Kenya from Kenya Demographic and Health Survey data of 2022”, how do you relate with the indicated objective in the abstract part? Even with that of indicated title? What is the difference between “Determinants” and “associated factors”?
   - The introduction lacks the prevalence of depression in Africa particularly in Keny and lacks relevant literature to establish the rationale.
   - Overall, the introduction is poorly written.

**Objectives:**

3. The manuscript does not clearly state specific study objectives, which is a critical omission. Specific objectives should be explicitly defined, including any prespecified hypotheses.

**Methods:**

4. The manuscript accurately presents the key elements of the study design, appropriately considering it as a secondary data analysis.
5. The description of the setting, locations, and data collection dates is correctly provided.
6. Since this is a secondary data analysis, the absence of eligibility criteria and participant selection details is acceptable so I didn’t see such thing.
7. The dependent and independent variables are not well-defined. How do you measure your dependent variable? Even if, the dependent variable was used from the KDHS definitions as reported in 2022, you should have established its measurement with relevant literature or you can operationalize it as well. Mention its indictor.
8. While all independent variables of the study indicated, sources of data for each variable and their method of data collection are not mentioned. You should have to describe each independent variable.
9. Why Stata version 14? Why not 17?
10. The manuscript explains how quantitative variables were handled in the analyses, including the use of different analytical techniques. However, irrelevant sentences types are described (Lines 126-127), which should be removed.
11. The statistical methods, including multilevel logistic regression to control for potential confounding variables, are well-described. However, it is not indicated how missing data were analysed.

**Results:**

12. There are several issues in the results section:

- Line 170:” deviation” how do you relate with that of deviance?
- The reference error "Error! Reference source not found" needs clarification.
- Missing data in baseline variables should be explained, along with the methods used to address the missing data.
- The manuscript should clarify the model fit statistics referenced in the text (Line 376). It is not clear how someone knows whether the given result is COR or AOR.
- While the third model contained community-level variables (Residence: lines 135-136), it is not statistically significant at 5% level of significance as presented in Table 3. So, do you think that multilevel analysis is suitable or appropriate statistical method for the given data set? Is it relevance?

**Discussion:**

13. The 'Discussion' section can be generally divided into 3 separate paragraphs. 1) Introductory paragraph/rationale of the study, 2) Intermediate paragraphs/compare and contrast with the most recent and relevant literature, 3) Concluding paragraph/indicating future directions. The introductory paragraph contains the main idea of performing the study question.
- While the manuscript mentions the large sample size as a strength indicating there is adequate power to detect the true effect of the independent variable. However, it does not provide a power calculation to support this claim.
- Some limitations are discussed, but the limitation related to the 24-hour recall for bias is not mentioned and there is potential social desirability bias that could impact on the results. Discuss finding should be interpreted with caution.

**Recommendation**

This comprehensive report outlines the issues identified throughout the manuscript, with specific line references and requires substantial major revisions to address these concerns and enhance the clarity, relevance, and presentation of results. After making the necessary major revisions with re-analysis of the data, a re-evaluation is recommended for considering publication.
